# The mediating role of serum 25-hydroxyvitamin D on the association between reduced sensitivity to thyroid hormones and periodontitis in Chinese euthyroid adults

**DOI:** 10.3389/fendo.2024.1456217

**Published:** 2024-10-30

**Authors:** Hao Yang, Yayun Lu, Lina Zhao, Yufeng He, Yuecheng He, Dong Chen

**Affiliations:** ^1^ Department of Stomatology, Health Examination Center of Shanghai Health and Medical Center, Huadong Sanatorium, Wuxi, China; ^2^ Nursing Department, Health Examination Center of Shanghai Health and Medical Center, Huadong Sanatorium, Wuxi, China; ^3^ Department of Stomatology, The affiliated Hospital of Inner Mongolia Medical University, Inner Mongolia Medical University, Hohhot, China; ^4^ Department of Oral and Maxillofacial Implantology, Shanghai PerioImplant Innovation Center, Shanghai Ninth People’s Hospital, Shanghai Jiao Tong University School of Medicine, Shanghai, China; ^5^ College of Stomatology, Shanghai Jiao Tong University, National Center for Stomatology, National Clinical Research Center for Oral Diseases, Shanghai Key Laboratory of Stomatology, Shanghai Research Institute of Stomatology, Shanghai, China; ^6^ Department of Endodontics, Shanghai Stomatological Hospital & School of Stomatology, Fudan University, Shanghai, China; ^7^ Shanghai Key Laboratory of Craniomaxillofacial Development and Diseases, Fudan University, Shanghai, China

**Keywords:** sensitivity to thyroid hormones, periodontitis, euthyroid, 25(OH)D, mediation analysis

## Abstract

**Aim:**

Thyroid dysfunction is closely associated with periodontitis. We aim to explore the association between sensitivity to thyroid hormones (THs) and periodontitis and to investigate the mediating role of serum 25-hydroxyvitamin D[25(OH)D] in this relationship in Chinese euthyroid populations.

**Methods:**

This population-based retrospective study included 2,530 euthyroid participants. Central sensitivity to THs was assessed by the thyroid feedback quantile-based index (TFQI), parametric thyroid feedback quantile-based index (PTFQI), thyrotrophic thyroxine resistance index (TT4RI) and thyroid-stimulating hormone index (TSHI), while FT3/FT4 was evaluated to assess peripheral sensitivity. Multivariable regression analysis and restricted cubic spline were performed to explore the association between sensitivity to THs and periodontitis. Threshold effect and subgroup analysis were also conducted. Mediation analysis was performed to estimate direct and indirect effects through 25(OH)D.

**Results:**

Multivariable regression analysis indicated that central sensitivity to THs indices(per SD increase) were positively associated with periodontitis risk [TFQI: OR=1.19,95% CI (1.09, 1.31); PTFQI: OR=1.22, 95% CI(1.12,1.34); TSHI: OR=1.36, 95% CI (1.21,1.52); TT4RI: OR=1.43, 95% CI (1.25,1.63)](all *P value*<0.001). TT4RI only had a non-linear relationship with periodontitis in euthyroid participants. Subgroup analysis showed that no significant correlations were founded among those aged over 65 years or with hypertension/diabetes. Mediation analysis revealed that the proportions mediated by 25(OH)D on the association of TFQI, PTFQI,TSHI, TT4RI and periodontitis risk were 16.37%, 16.43%, 9.93% and 10.21%, respectively.

**Conclusions:**

Impaired central sensitivity to THs is positively associated with periodontitis in euthyroid and serum 25(OH)D might be one of its biological mechanisms.

## Introduction

Periodontitis, a prevalent health concern, is defined by the deterioration of the soft and hard tissues surrounding the teeth, resulting from the dysregulation of the host immune response triggered by subgingival microorganisms (1). The primary cause of this condition is bacteria residing in dental plaque. Moreover, the susceptibility of the host plays a critical role in both the initiation and progression of periodontitis (2). Affecting 10–15% of individuals worldwide, periodontitis has been associated with several chronic inflammation-driven disorders through epidemiological studies, significantly impacting their quality of life (3). Furthermore, its detrimental effects on various systemic health conditions like respiratory disease, diabetes, and cardiovascular disease contribute to a heavy global medical burden and present a significant public health challenge (4–6). Recent decades have seen a growing interest in the potential relationships between periodontal disease and systemic disorders. Numerous studies have shown a positive correlation between metabolic disorders, such as diabetes, metabolic syndrome, and osteoporosis (7, 8).

The thyroid, the largest endocrine organ in the body, is frequently targeted by autoimmune diseases. A recent study found a significant association between high community periodontal index (CPI) and abnormalities in thyroid function tests (9). Additionally, A scoping review has shown a positive relationship between hypothyroidism and periodontitis (10). Study has also demonstrated that levels of thyroid stimulating hormone (TSH) are independently related to the development of periodontitis (11). However, solely measuring serum TSH, free triiodothyronine (FT3), and free thyroxine (FT4) levels in euthyroid individuals is not sufficient for evaluating thyroid function status. It is important to consider that thyroid hormone homeostasis may not be stable even if these indicators are within the normal range (12).

The first proposal of a new thyroid functional entity called sensitivity to THs was made by Refetoff et al. This entity considers both FT4 and TSH levels, and is defined by high levels of FT4 and FT3 along with normal or slightly elevated TSH (13). The resistance to thyroid hormones syndrome is characterized by high levels of both FT4 and TSH, indicating issues with energy balance. To assess the sensitivity to thyroid hormones, researchers have proposed various indices, such as the thyroid feedback quantile-based index (TFQI) and parametric thyroid feedback quantile-based index (PTFQI) (12, 14), as well as the thyrotrophic thyroxine resistance index (TT4RI) and thyroid-stimulating hormone index (TSHI) (15, 16), to quantify the central sensitivity to THs. Besides, FT3/FT4 was evaluated to represent peripheral sensitivity to THs. Previous research has shown a positive correlation between sensitivity to thyroid hormones and various health conditions such as prediabetes, reduced glomerular filtration, and high levels of remnant cholesterol (17–19). Likewise, recent study illustrated that impaired thyroid hormone sensitivity correlates with decreased vitamin D levels among euthyroid subjects (20). Moreover, results of another cross-sectional study identified significant relationships between periodontitis with 25(OH)D metabolites (21), which indicated that serum 25(OH) D may play a role in the association between sensitivity to THs and periodontitis risk.

Therefore, as scarce study had ever explored the impact of sensitivity to THs on periodontitis, this study aims to investigate the association between central and peripheral sensitivity to THs and periodontitis in euthyroid populations, and further quantify the direct and indirect associations by serum 25(OH) D, and to provide clinical evidence of maintaining periodontal health in euthyroid subjects.

## Methods

### Study population and design

**Figure 1:**
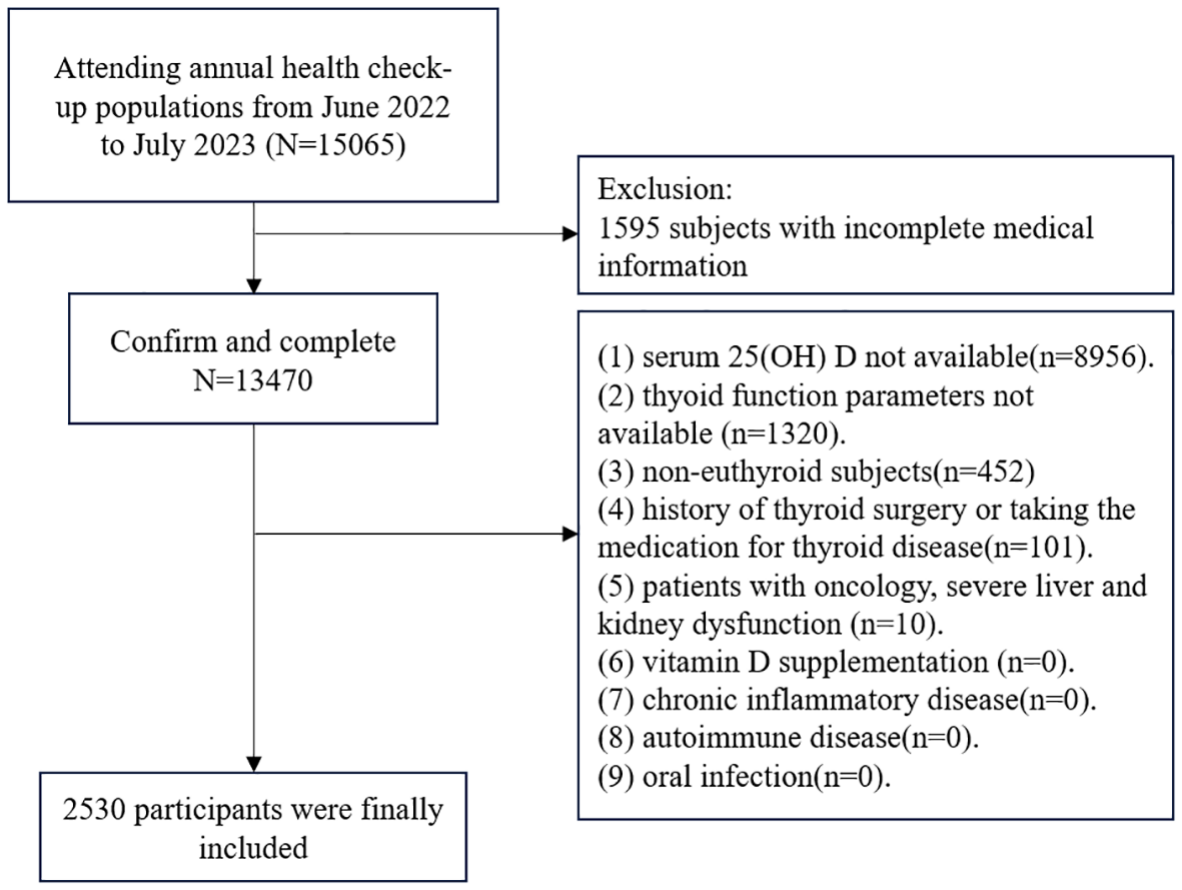
Flowchart of study participants.

The study involved individuals who were over 18 years old and had participated in annual health examinations at the health check-up center of Huadong sanatorium. Initially, a total of 15,065 adults were included in this retrospective study. Participants were excluded if they met the following criteria: (1) incomplete medical information; (2) serum 25-hydroxyvitamin D not available; (3) blood parameters of thyroid function test not available; (4) non-euthyroid subjects; (5) history of thyroid surgery or taking the medication for thyroid disease; (6) patients with oncology, severe liver and kidney dysfunction; (7) vitamin D supplementation; (8) chronic inflammatory disease; (9) autoimmune disease; (10) oral infection. After excluding these individuals, a total of 2,530 participants were included in the study (**Figure 1**), consisting of 1,092 females and 1,438 males aged between 18 and 90 years. Adhering to the principles of the Declaration of Helsinki, this retrospective study received approval from the Health Examination Center of Huadong sanatorium. Patient data was anonymized to ensure confidentiality, and statistical analysis was conducted in a secure manner for scientific research. Consequently, the necessity for informed consent was exempted.

### Handling of missing data

In our analysis, we managed missing data by employing multiple imputation, which involves estimating missing values from a distribution informed by the observed ones. We applied chained equations for this imputation, capturing the imputation uncertainty in our analysis. To reinforce the validity of our results, sensitivity analyses were performed across various imputation scenarios. These analyses verified the consistency of our conclusions despite the presence of missing data, attesting to the dependability of our study’s findings.

### Sample size estimation

To confirm that our analysis had sufficient statistical power, we performed a power calculation utilizing PASS 2021 software. Given the 30% occurrence rate of periodontitis, the calculation indicated that a sample size of 1,329 individuals would be necessary to identify a significant effect at an alpha level of 0.05 with 80% power. Anticipating a potential 10% non-response rate, we adjusted the sample size to 1,462. Finally, our study included 2,530 participants, which not only meets but also surpasses the projected sample size. This ensures ample power to detect meaningful associations within our data.

### Assessment of covariates

A standard questionnaire was used to collect demographic characteristics such as age, gender, and cigarette/alcohol use. Smoking was defined as consuming three or more cigarettes daily for a year, while alcohol consumption was defined as drinking at least three times a week for twelve months. Fasting venous blood samples were collected from all participants following a 12-hour overnight fast. Levels of fasting plasma glucose (FPG), triglycerides (TG), total cholesterol (TC), high-density lipoprotein cholesterol (HDL-C), low-density lipoprotein cholesterol (LDL-C), neutrophils (NE), and lymphocytes (LY) were measured using an automatic hematology analyzer. Strict quality control procedures were adhered to in the laboratory.

Additionally, we collected health-related information, including whether individuals had a previous diagnosis of hypertension or diabetes, and if they were currently taking any medications. Diabetes was defined as having FBG levels≥7.0 mmol/L, being prescribed insulin or oral hypoglycemic agents, or self-reporting a history of the condition (22). Hypertension was determined by having SBP≥140 mmHg or DBP≥90mmHg, and currently using antihypertensive medications (23). Dyslipidemia was defined as having TC≥5.2 mmol/L or LDL-C≥3.4 mmol/L or HDL-C<1.0 mmol/L or TG≥1.7 mmol/L (24), while electrochemiluminescence immunoassay method was used to measure the concentrations of 25 (OH)D, TSH, FT3, and FT4 according to the manufacturer’s instructions. The reference ranges for FT3, FT4, and TSH were 3.10~6.80 pmol/L, 12.00~22.00 pmol/L, and 0.27~4.20mIU/L, respectively. Euthyroid was defined as having serum TSH and FT4 levels within the normal ranges and not using thyroid hormone medication.

The physical examination involved taking measurements of height, weight, and blood pressure. BMI was determined by dividing weight in kilograms by height in meters squared. Systolic and diastolic blood pressure were assessed on the right arm using a sphygmomanometer after a minimum of 5 minutes of rest, and the average of two readings was calculated.

### Ascertainment of periodontitis

The periodontal examination was conducted by experienced dentists on each participant. The following teeth were examined to assess overall periodontal health: maxillary right first molar, maxillary left central incisor, maxillary left first premolar, mandibular left first molar, mandibular right central incisor, mandibular right first premolar. If any of these teeth were missing, a substitute tooth was chosen as recommended by Fleiss et al. (25). Periodontal parameters such as probing depth (PD), clinical attachment level (CAL), and bleeding on probing (BOP) were measured at 6 points (mesio-buccal, mid-buccal, disto-buccal, disto-lingual, mid-lingual, mesio-lingual) around each tooth using a periodontal probe. The diagnosis of periodontitis was made according to the clinical definition provided by the Centers for Disease Control and Prevention in collaboration with the American Academy of Periodontology for population-based surveillance (CDC/AAP), with no periodontitis diagnosed when there was no evidence of mild, moderate, or severe periodontitis (26).

### Thyroid hormone sensitivity indices

Thyroid Feedback Quartile-Based index (TFQI), parametric thyroid feedback quantile-based index (PTFQI), TSH index (TSHI), and Thyrotroph T4 Resistance Index (TT4RI) were allocated to evaluate the participants’ central sensitivity to thyroid hormones with the following formulas (15, 27): TFQI =cumulative distribution function (cdf) FT4 − (1 − cdf TSH); PTFQI = φ((FT4-μFT4)/σFT4)- (1-φ ((ln TSH-μln TSH)/σlnTSH)), where μfT4 = 16.12, σfT4 = 1.92, μln TSH=0.62, and σln TSH=0.44. TSHI = ln TSH (mIU/L) + 0.1345 × FT4 (pmol/L). For TFQI and PTFQI, negative values indicated higher central sensitivity and positive values indicated lower central sensitivity to the change of FT4. TT4RI = FT4 (pmol/L) × TSH (mIU/L). For TSHI and TT4RI, higher values indicated lower central sensitivity to thyroid hormones. FT3 to FT4 ratio (FT3/FT4) was used to assess peripheral thyroid sensitivity. For FT3/FT4, higher values indicate higher peripheral sensitivity to thyroid hormones.

### Statistical analysis

Statistical analyses were conducted using SPSS 23.0 and R software (version 4.1). The normality of variables was assessed using the Kolmogorov-Smirnov test. Normally distributed variables were presented as mean (standard deviation), skewed variables as median [interquartile range], and categorical variables as frequencies (proportions). Group comparisons for continuous variables were performed using either Student’s t-test or Mann Whitney U test, while categorical variables were compared using the chi-square test. The association between sensitivity to THs and the baseline characteristics of the euthyroid participants was evaluated using Spearman’s coefficient correlation analysis (r). Multivariable logistic regression analysis was performed to evaluate the associations of periodontitis risk with per SD increase or quartiles of THs sensitivity indices using two models adjusting for possible confounding factors. Model 1 was adjusted for sex, age, smoking and drinking as covariates, while Model 2 additionally adjusted for waist circumference, body mass index, diabetes, hypertension, dyslipidemia, NLR and 25(OH)D. We further used restricted cubic spline with four knots located at the 5th, 35th, 65th and 95th percentiles of the exposure distribution to assess the adjusted non-linear relationship between thyroid hormones sensitivity indices and periodontitis risk, and we also analyzed the threshold effect and saturation effect on it. The model 1 in the threshold analysis is a linear relationship, and the effect size is obtained; model 2 is a non-linear relationship, and the effect size of the inflection point and the segment is obtained. Th e log-likelihood ratio test was used to verify the difference between model 1 and model 2. Subgroup analysis was used to assess whether the correlations between central sensitivity to THs and periodontitis were stable across cohorts stratified by gender, age, BMI, WC, smoking, drinking, hypertension, diabetes, and dyslipidemia. Furthermore, to verify the stability of the results, a sensitivity analysis was conducted, excluding participants who had poor oral hygiene habits. We also conducted a mediation analysis to assess the direct and indirect associations between impaired sensitivity to thyroid hormones and periodontitis via serum 25(OH)D using an available R package named “mediation”. A significance level of 0.05 was used for statistical tests.

## Results

### Characteristics of the study population

**Table 1** provides an overview of the baseline characteristics of the participants in this study. A total of 2,530 euthyroid individuals were included, with an average age of 50 ± 12 years, consisting of 1,092 females and 1,438 males. Among these participants, 1,583 individuals (37.4%) met the criteria for periodontitis as per CDC/AAP guidelines. Participants with periodontitis were more likely to be male, older, smokers, and alcohol drinkers (P<0.001). They also had a higher prevalence of metabolic conditions such as diabetes and dyslipidemia (P<0.05). Additionally, individuals with periodontitis had higher levels of systolic blood pressure (SBP), diastolic blood pressure (DBP), fasting blood glucose (FBG), triglycerides (TG), and 25-hydroxyvitamin D (25(OH)D), and lower levels of high-density lipoprotein cholesterol (HDL-C) compared to healthy controls. In addition, the levels of central thyroid hormones sensitivity indices (PTFQI, TFQI, TSHI, TT4RI) and TSH, were significantly higher among participants with periodontitis than non-periodontitis group (P<0.01), and no significant difference was observed in peripheral sensitivity to THs (FT3/FT4) levels between individuals with periodontitis and those without (P=0.170). Additionally, when stratified analyses were conducted on periodontitis prevalence among sensitivity to THs quartiles (**Supplementary Figure S1**), the results showed that periodontitis prevalence increased with higher quartiles of PTFQI, TFQI, TSHI, and TT4RI, except for FT3/FT4 (all P _for trend_<0.05). In males, periodontitis prevalence increased as the quartiles of TSHI and TT4RI rose. For females, periodontitis prevalence also increased as the quartiles of PTFQI, TSHI, and TT4RI increased. Among individuals aged 18-44 years or 45-65 years, periodontitis prevalence rose with higher quartiles of PTFQI, TFQI, TSHI, and TT4RI. However, there was no significant increasing trend in periodontitis prevalence with higher quartiles of all sensitivity to THs indices among those aged over 65 years (all P _for trend_ >0.05).

**Table 1:** Baseline characteristics of all participants.

Characteristic	Overall, N = 2,530	Non-periodontitis N = 1,583	Periodontitis N = 947	p-value
Sex, n (%)				<0.001
Female	1,092 (43.2%)	737 (46.6%)	355 (37.5%)	
Male	1,438 (56.8%)	846 (53.4%)	592 (62.5%)	
Age, years	50 ± 12	48 ± 12	53 ± 11	<0.001
BMI, kg/m2	24.5 ± 3.2	24.3 ± 3.3	24.8 ± 3.1	<0.001
WC, cm	83 ± 10	82 ± 10	84 ± 9	<0.001
Smoking, n (%)	626 (24.7%)	358 (22.6%)	268 (28.3%)	0.001
Drinking, n (%)	446 (17.6%)	239 (15.1%)	207 (21.9%)	<0.001
SBP, mmHg	122 ± 16	121 ± 16	124 ± 16	<0.001
DBP, mmHg	73 ± 11	73 ± 10	74 ± 11	<0.001
FBG, mmol/L	5.48 ± 1.10	5.39 ± 0.97	5.63 ± 1.26	<0.001
diabetes, n (%)	174 (6.9%)	90 (5.7%)	84 (8.9%)	0.002
hypertension, n (%)	299 (11.8%)	174 (11.0%)	125 (13.2%)	0.096
TG, mmol/L	1.22 (0.89, 1.81)	1.19 (0.84, 1.77)	1.27 (0.93, 1.91)	<0.001
TC, mmol/L	4.83 (4.26, 5.39)	4.80 (4.27, 5.39)	4.86 (4.26, 5.42)	0.391
LDL-C, mmol/L	3.09 (2.58, 3.62)	3.05 (2.57, 3.61)	3.14 (2.60, 3.65)	0.089
HDL-C, mmol/L	1.29 (1.07, 1.55)	1.30 (1.08, 1.56)	1.25 (1.06, 1.51)	0.007
dyslipidemia, n (%)	938 (37.1%)	562 (35.5%)	376 (39.7%)	0.034
NLR	1.53 (1.20, 1.90)	1.53 (1.20, 1.90)	1.54 (1.20, 1.92)	0.513
FT4, pmol/L	15.90 (14.60, 17.30)	15.90 (14.60, 17.30)	15.80 (14.60, 17.30)	0.638
FT3, pmol/L	4.70 (4.33, 5.10)	4.69 (4.32, 5.09)	4.71 (4.36, 5.12)	0.200
TSH, uIU/ml	1.91 (1.37, 2.61)	1.81 (1.30, 2.56)	2.02 (1.50, 2.69)	<0.001
PTFQI	-0.02 (-0.23, 0.18)	-0.04 (-0.24, 0.17)	0.01 (-0.20, 0.21)	<0.001
TFQI	-0.08 (-0.28, 0.13)	-0.09 (-0.29, 0.11)	-0.06 (-0.27, 0.16)	0.005
TSHI	2.79 (2.42, 3.12)	2.74 (2.38, 3.08)	2.85 (2.51, 3.16)	<0.001
TT4RI	30 (22, 41)	29 (21, 40)	32 (24, 43)	<0.001
FT3/FT4 ratio	0.30 (0.27, 0.32)	0.29 (0.27, 0.32)	0.30 (0.27, 0.33)	0.170
25(OH) D, ng/ml	16.10 (12.30, 20.90)	16.90 (12.80, 21.40)	15.10 (11.60, 19.80)	<0.001

Continuous variables are presented as mean ± standard deviation or median (interquartile) with number (proportion, %) for categorical variables. P values among groups are calculated by one-way ANOVA or Kruskal-Wallis H tests for continuous variables, Chi-square test for categorical variables. WC, waist circumference; BMI, body mass index; SBP, systolic blood pressure; DBP, diastolic blood pressure; FPG, fasting plasma glucose; TG, triglycerides; TC, total cholesterol; HDL-C, high-density lipoprotein cholesterol; LDL-C, low-density lipoprotein cholesterol; NLR, neutrophil to lymphocyte ratio; FT3, free triiodothyronine; FT4, free thyroxine; TSH, thyroid-stimulating hormone; PTFQI, parametric thyroid feedback quantile-based index; TFQI, thyroid feedback quantile-based index; TSHI, TSH index; TT4RI, thyrotropin thyroxine resistance index; FT3/FT4, free triiodothyronine to free thyroxine ratio; 25(OH) D, 25-hydroxyvitamin D.

### Correlation between all sensitivity to THs indices and baseline characteristics

**Supplementary Table S1** shows the correlations between all sensitivity to THs and baseline characteristics of the participants. PTFQI was positively correlated with DBP, but negatively correlated with age (P<0.01). TFQI was positively correlated with SBP, DBP and FBG, but negatively correlated with age (P<0.05). TSHI was positively correlated with TC and LDL-C, but negatively correlated with age, and NLR (P<0.05). TT4RI was positively correlated with TC and LDL-C, but negatively correlated with NLR (P<0.01). FT3/FT4 ratio was positively correlated with WC, BMI, SBP, DBP, and TG, but negatively correlated with TC, HDL-C, and NLR (P<0.05).

### Associations of central sensitivity to THs indices with periodontitis

As shown in **Table 2**, with regard to continuous variables in multivariable logistic regression analysis, after adjustment for potential confounders, central sensitivity to THs indices(per SD increase) were positively associated with periodontitis risk [TFQI: OR=1.19,95% CI (1.09, 1.31); PTFQI: OR=1.22, 95% CI(1.12,1.34); TSHI: OR=1.36, 95% CI (1.21,1.52); TT4RI: OR=1.43, 95% CI (1.25,1.63)](all P value <0.001). Besides, compared with the lowest quartile of central sensitivity to THs indices, those in the highest quartile showed increasing positive association with periodontitis risk after adjustment [TFQI: OR=1.41,95% CI (1.11,1.79); PTFQI: OR=1.57, 95% CI (1.24,2.00); TSHI: OR=1.90, 95% CI(1.50,2.42); TT4RI: OR=1.94, 95% CI(1.53,2.48)](all P value <0.001).

**Table 2:** Multivariable regression analysis of Association between quartiles of central sensitivity to THs and periodontitis risk.

	Crude			Model 1			Model 2		
	OR	95% CI	p-value	OR	95% CI	p-value	OR	95% CI	p-value
TFQI (+1 SD)	1.14	1.05, 1.25	0.003	1.19	1.09, 1.31	<0.001	1.19	1.09, 1.31	<0.001
Q1	Ref.			Ref.			Ref.		
Q2	0.98	0.77, 1.23	0.831	1.01	0.80, 1.28	0.933	1.02	0.80, 1.29	0.886
Q3	1.02	0.81, 1.29	0.844	1.1	0.86, 1.39	0.449	1.1	0.87, 1.39	0.443
Q4	1.27	1.01, 1.59	0.038	1.41	1.11, 1.78	0.005	1.41	1.11, 1.79	0.004
PTFQI (+1 SD)	1.17	1.07, 1.27	<0.001	1.22	1.12, 1.33	<0.001	1.22	1.12, 1.34	<0.001
Q1	Ref.			Ref.			Ref.		
Q2	1.09	0.86, 1.38	0.465	1.07	0.84, 1.35	0.602	1.08	0.85, 1.37	0.549
Q3	1.29	1.03, 1.63	0.029	1.37	1.08, 1.73	0.01	1.37	1.08, 1.74	0.009
Q4	1.44	1.15, 1.81	0.002	1.56	1.23, 1.98	<0.001	1.57	1.24, 2.00	<0.001
TSHI (+1 SD)	1.29	1.16, 1.44	<0.001	1.35	1.21, 1.51	<0.001	1.36	1.21, 1.52	<0.001
Q1	Ref.			Ref.			Ref.		
Q2	1.34	1.06, 1.69	0.014	1.36	1.07, 1.73	0.012	1.37	1.07, 1.74	0.011
Q3	1.45	1.15, 1.83	0.002	1.57	1.23, 2.00	<0.001	1.57	1.23, 1.99	<0.001
Q4	1.74	1.38, 2.19	<0.001	1.91	1.50, 2.42	<0.001	1.9	1.50, 2.42	<0.001
TT4RI (+1 SD)	1.36	1.20, 1.55	<0.001	1.43	1.25, 1.63	<0.001	1.43	1.25, 1.63	<0.001
Q1	Ref.			Ref.			Ref.		
Q2	1.53	1.21, 1.94	<0.001	1.58	1.24, 2.01	<0.001	1.58	1.24, 2.02	<0.001
Q3	1.6	1.26, 2.02	<0.001	1.7	1.34, 2.17	<0.001	1.7	1.34, 2.17	<0.001
Q4	1.8	1.43, 2.28	<0.001	1.95	1.53, 2.48	<0.001	1.94	1.53, 2.48	<0.001

OR, Odds Ratio; CI, Confidence Interval.

Model 1: adjusted for sex, age, smoking, and drinking.

Model 2: adjusted for sex, age, smoking, drinking, WC, BMI, 25(OH)D, NLR, diabetes, hypertension, and dyslipidemia.

### Smooth curve fitting and threshold effect analysis

**Figure 2:**
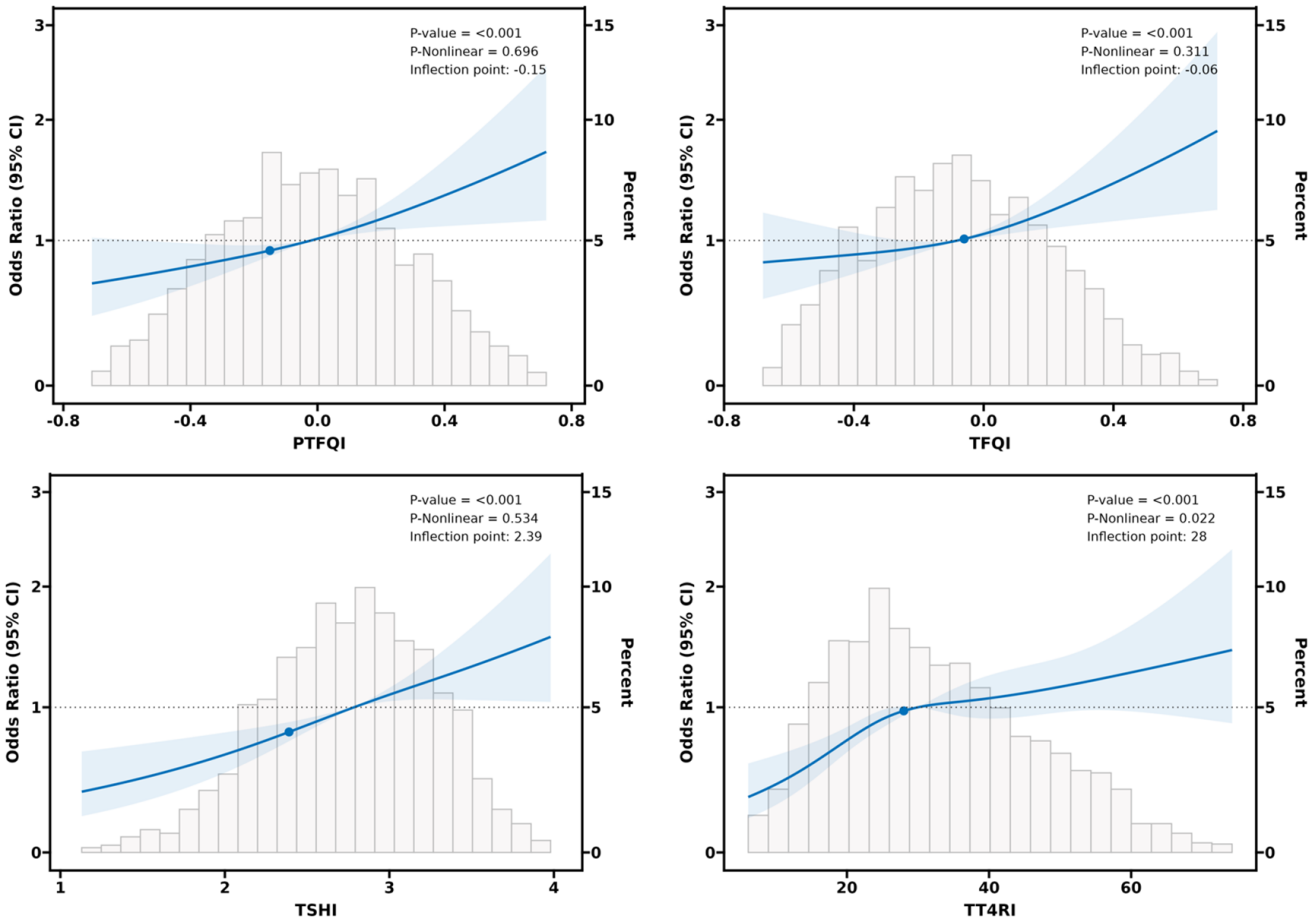
Dose-relationship between central sensitivity to THs indices and periodontitis.

According to the results of regression analysis, we explored whether the central sensitivity to THs indices and the likelihood of periodontitis were non-linear and verified with a threshold effect through smooth curve fitting analysis, and the results are shown in **Figure 2**. A non-linear relationship was observed between TT4RI and periodontitis (P nonlinear=0.022), while there was a linear association between PTFQI, TFQI, and TSHI and periodontitis (all P nonlinear>0.05), which was in accordance with the results of the threshold effect model (**Table 3**). Moreover, regarding to TT4RI, the log-likelihood ratio test *P*=0.001, and indicates that a two-piece linear regression model should be used to fit the model, the infection point was calculated to be 28 by a two-segment linear regression model and recursive algorithm. Confidence intervals for thresholds were determined by the Bootstrap resampling method. The results indicated that there was a threshold effect between TT4RI and periodontitis, on the left side of the infection point (TT4RI=28), the positive relationship between TT4RI and periodontitis was more significant, with an effect value of [OR=1.05, 95% CI: (1.03,1.07)], revealing that each unit increase of TT4RI was associated with a 5% increasing in periodontitis risk (*P*<0.001).However, on the right side of the infection point, the effect size and 95% CI was 1.01 (0.97, 1.02), which means that each unit increase of TT4RI was not associated with periodontitis risk (P=0.256).

**Table 3:** Threshold and saturation effect analysis of central sensitivity to THs on periodontitis.

	Adjusted OR (95% CI) for Periodontitis, *P*-value			
	TFQI	PTFQI	TSHI	TT4RI
Model 1				
Fitting model by standard linear regression	1.81(1.33,1.41), <0.001	1.92(1.42, 2.54), <0.001	1.62(1.31, 1.92), <0.001	1.02(1.01, 1.03), <0.001
Model 2				
Fitting models by two-piecewise linear regression				
Inflection point	-0.06	-0.15	2.39	28
<Inflection point	1.33 (0.72, 2.41), 0.369	1.51 (0.71, 3.22), 0.309	1.72 (1.00, 2.84), 0.040	1.05 (1.03, 1.07), <0.001
> Inflection point	2.34 (1.32, 4.00) 0.004	2.13 (1.42, 3.23), <0.001	1.63 (1.22, 2.09), <0.001	1.01 (0.97, 1.02), 0.256
*P* for log-likelihood ratio test	0.279	0.538	0.788	0.001

Adjusted for sex, age, smoking, drinking, WC, BMI, 25(OH)D, NLR, diabetes, hypertension, and dyslipidemia.

### Subgroup analysis

**Figure 3:**
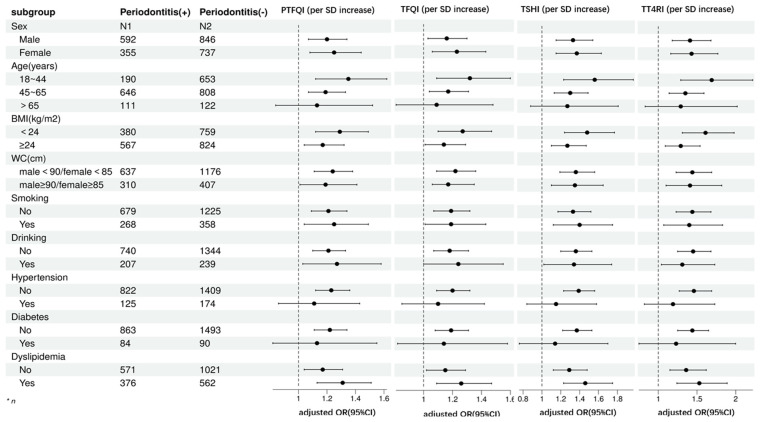
Subgroup analysis.

We conducted a subgroup analysis to assess the reliability of our findings stratified by gender, age, BMI, WC, smoking, drinking, hypertension, diabetes, and dyslipidemia after adjustment for confounders. The detailed subgroup results were outlined in **Figure 3**. The positive relationship of all central sensitivity to THs indices and periodontitis was stable across sex (male or female), age (18~44 years or 45~65 years), BMI(≥24.0kg/m2 or <24kg/m2), WC(male≥90cm and female≥85cm,or male<90cm and female<85cm), smoking(Yes or No), drinking(Yes or No) and dyslipidemia(Yes or No). Nevertheless, there was no significantly positive association between sensitivity to THs indices and periodontitis among those with hypertension and diabetes, or aged over 65 years (all P value >0.05).

### Sensitivity analysis

Due to the heightened susceptibility to periodontitis associated with detrimental oral hygiene practices—like daily dental cleansing not exceeding once, frequent intake of sugar-laden drinks, and a one-year gap since the last oral health examination—a total of 825 participants exhibiting these traits were eliminated from the sensitivity analyses. In alignment with the primary study, we examined the association between central sensitivity to thyroid hormones (THs) and the likelihood of periodontitis employing three multivariable regression models. The findings consistently demonstrated a robust, positive correlation between TH sensitivity and the risk of periodontitis (**Supplementary Table S2**).

### Mediation analysis through 25(OH)D

**Figure 4:**
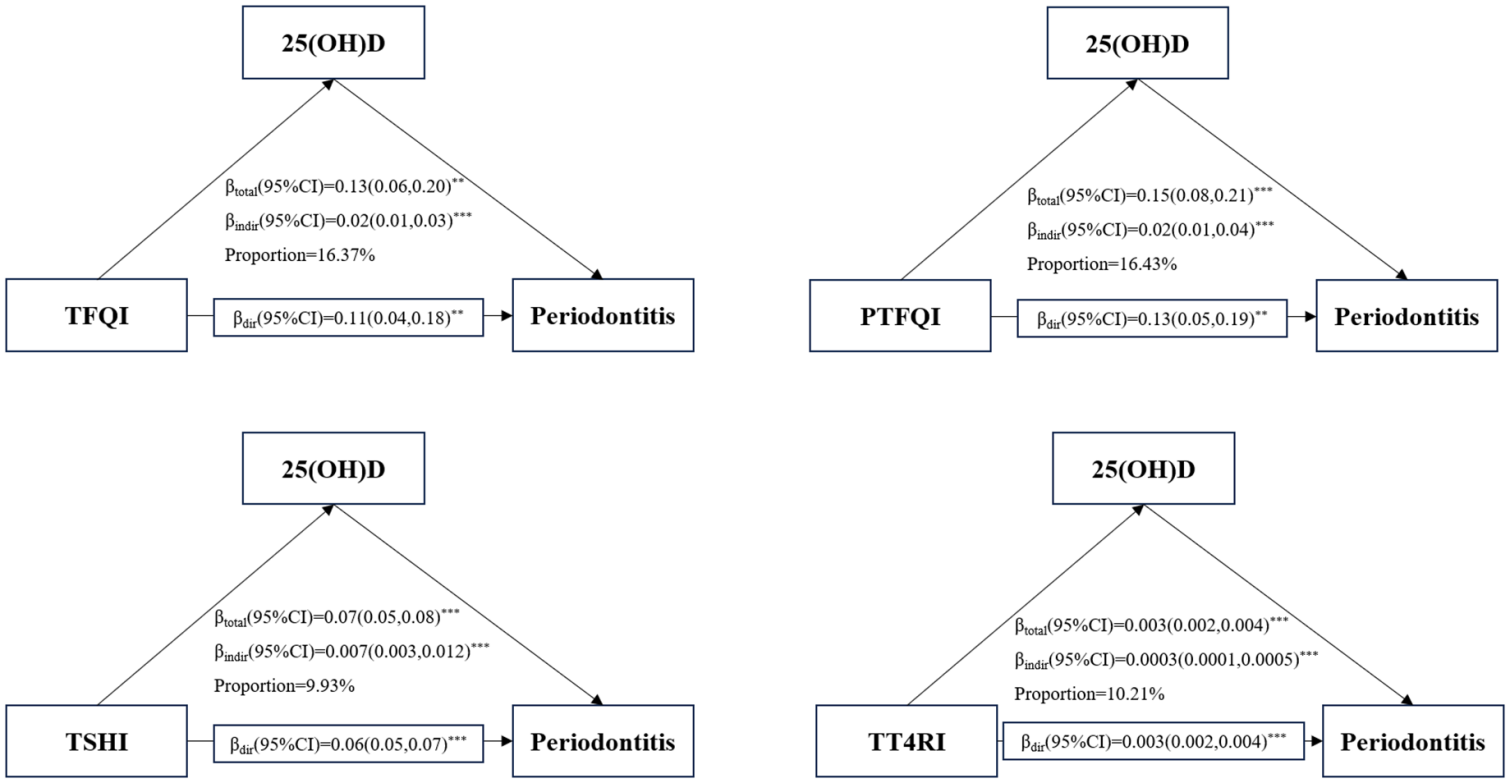
Mediation analyses of the association between central THs sensitivity indices and periodontitis through 25(OH)D. **P-value<0.01; ***P-value<0.001.

**Figure 4** presents that mediating effect of 25(OH)D on the association between central sensitivity to THs indices and periodontitis risk. This study revealed a significant indirect effect of TFQI, PTFQI,TSHI, TT4RI and periodontitis risk (all P value<0.001). The proportions mediated by 25(OH)D on the association of TFQI, PTFQI,TSHI, TT4RI and periodontitis risk were 16.37%, 16.43%, 9.93% and 10.21%, respectively. Additionally, the results also show a significantly direct and indirect coefficients between four indices of central sensitivity to thyroid hormones and periodontitis through 25(OH)D(all P value<0.001).

## Discussion

To the best of our knowledge, this cross-sectional study is the first to investigate the association of sensitivity to THs and periodontitis in euthyroid populations. Our study found that decreased central sensitivity to THs markers (elevated TFQI, PTFQI, TSHI, and TT4RI) were related to a higher risk of periodontitis in Chinese euthyroid individuals. Subgroup analysis showed consistent positive associations across all groups except for those over 65 years old or with hypertension/diabetes. We also discovered a non-linear relationship between TT4RI and periodontitis, while linear relationships were found between PTFQI, TFQI, TSHI, and periodontitis. Additionally, our research revealed that serum 25(OH)D played a role in mediating the connection between central sensitivity to THs and periodontitis, suggesting that addressing low levels of 25(OH)D could impact the development of periodontitis in individuals with normal thyroid function.

Thyroid hormones play a crucial role in regulating energy metabolism. Early stages of thyroid dysfunction often go unnoticed, leading to delayed detection and underestimation of its severity. While subclinical thyroid dysfunction with normal fT4 levels has not received much attention in the past, recent reports have shown that asymptomatic thyroid dysfunction can increase the risk of various diseases such as cardiovascular disease, bone fractures, and depressive mood disorders (9). It is important to closely monitor and assess the presence of asymptomatic thyroid dysfunction and its associated risk factors. Studies have indicated a link between thyroid dysfunction and poor oral health, particularly in relation to periodontitis. Research has shown a positive association between hypothyroidism and periodontitis, suggesting that thyroid dysfunction may contribute to the development of this oral health condition (28). However, further studies have found that hyperthyroidism may also play a role in promoting inflammation in periodontitis (29). The inconsistency in these findings highlights the complexity of the relationship between the thyroid system and periodontitis, indicating that simply measuring TSH or thyroid hormone levels may not be sufficient to fully understand this connection. Comprehensive indices that consider multiple factors are needed to accurately assess the regulation of thyroid hormone homeostasis in relation to periodontitis.

The regulation of TSH and FT4 in the hypothalamic-pituitary-thyroid axis is controlled by a negative feedback mechanism. High levels of both TSH and FT4 indicate acquired resistance to thyroid hormones, a newly recognized clinical condition. A thyroid hormone sensitivity index (TFQI) was proposed in 2019 by Laclaustra et al. to assess the risk of metabolic syndrome, diabetes, and diabetes-related mortality in individuals with normal thyroid function (27). Previous research has shown that impaired sensitivity to thyroid hormones can lead to various health issues such as diabetes, hypertension, and renal dysfunction (12, 30). However, there is limited research on whether impaired sensitivity to thyroid hormones is linked to periodontitis. Therefore, our study aimed to investigate this potential relationship. Our study found a correlation between reduced sensitivity to THs and an increased risk of periodontitis in euthyroid individuals. Elevated levels of TSH in those with THs resistance may contribute to inflammation associated with obesity by promoting the production of pro-inflammatory cytokines in fat tissue (31, 32). Additionally, TSH levels, in combination with leptin, can indirectly lead to insulin resistance by stimulating the synthesis of pro-inflammatory cytokines in fat cells (33). Insulin resistance has been shown to exacerbate the body’s inflammatory response to periodontal bacteria, hindering the healing process and leading to faster deterioration of oral health (34). Previous research has also indicated a causal relationship between periodontitis and hypertension, with improvements in endothelial function observed after periodontal treatment (35). Furthermore, older individuals showed a higher prevalence of periodontitis and tooth loss (36). However, our study did not find a significant association between sensitivity to THs and periodontitis in individuals with hypertension, diabetes, or those over 65 years old. This suggests that diabetes, hypertension, and aging play a larger role in the development of periodontitis, with thyroid function having less impact in euthyroid individuals.

Recent studies have shown a correlation between impaired thyroid hormone sensitivity and vitamin D deficiency in euthyroid adults (20, 37). A nationwide population-based study also found a link between vitamin D deficiency and a high prevalence of thyroid autoimmunity and dysfunction (38). Additionally, a mendelian randomization study revealed significant relationships between periodontitis and 25(OH)D metabolites, suggesting a potential role of serum 25(OH)D in the association between THs sensitivity and periodontitis (21). Our study further explored the mediation effect of 25(OH)D on this association, finding that 25(OH)D mediated 9.93-16.43% of the associations between central sensitivity to thyroid hormones and periodontitis. In a cross-sectional study, serum 25(OH)D levels were negatively correlated with FT3, FT4, and TSH levels in euthyroid adults (37), and our study also showed that participants with periodontitis had lower levels of serum 25(OH)D compared to those without periodontitis. Furthermore, a large-sample size study involving 11,017 participants demonstrated a significant reduction in TSH and thyroid hormone levels after 12 months of vitamin D supplementation (39). *In vitro* studies have demonstrated that calcitriol administration can have both inhibitory and stimulatory effects on thyroid-stimulating hormone (TSH) release (40). While calcitriol suppressed TSH-stimulated adenylyl cyclase activity and iodide uptake (41), it was also shown to increase TSH release in rat pituitary cells (42). These findings suggest that vitamin D may have both central and peripheral influences on TSH and thyroid hormone regulation, although further research is needed to fully understand the underlying mechanisms. One potential mechanism by which vitamin D may reduce the risk of periodontitis is through the induction of cathelicidin (43). The vitamin D pathway has been found to be present in human gingival fibroblasts and periodontal ligament cells, where it plays a crucial role in immune defense by activating the antimicrobial protein cathelicidin (44, 45). In individuals with serum 25(OH)D deficiency, decreased levels of cathelicidin have been observed in periodontal tissues affected by gingivitis and chronic periodontitis (46).

This study has several limitations that should be acknowledged. Firstly, the cross-sectional design of the study means that the observed relationship between sensitivity to THs and periodontitis may not imply causation. Future longitudinal studies are necessary to determine the temporal relationship between these factors. Additionally, it would be valuable to explore whether vitamin D supplementation can improve thyroid function and periodontitis. Secondly, the study only assessed the presence of periodontitis without considering the severity of the condition. This lack of information makes it challenging to determine if the association between THs sensitivity and periodontitis varies depending on the severity of the condition. Lastly, despite adjusting for various potential confounding variables, there may still be residual bias that was not accounted for in the study.

## Conclusions

This research suggests a positive association between reduced central sensitivity to THs and the occurrence of periodontitis among euthyroid individuals. Additionally, it highlights the potential mediating role of 25(OH)D in this relationship. However, further investigation through clinical studies is required to confirm these findings and assess their potential as an intervention strategy.

## Data Availability

The raw data supporting the conclusions of this article will be made available by the authors, without undue reservation.

## Supplementary material

Supplementary Figure 1 Comparison of periodontitis prevalence among quartiles of different sensitivity to THs indices among all participants and subgroups. * *P _for trend_* <0.05, ** *P _for trend_* <0.01, *** *P _for trend_* <0.001, ns no significance.
