# Supplementary figures and images for: The mediating role of serum 25-hydroxyvitamin D on the association between reduced sensitivity to thyroid hormones and periodontitis in Chinese euthyroid adults
Source: Front Endocrinol (Lausanne). 2024 Oct 30;15:1456217. doi: 10.3389/fendo.2024.1456217 (PMC11557418; doi:10.3389/fendo.2024.1456217)

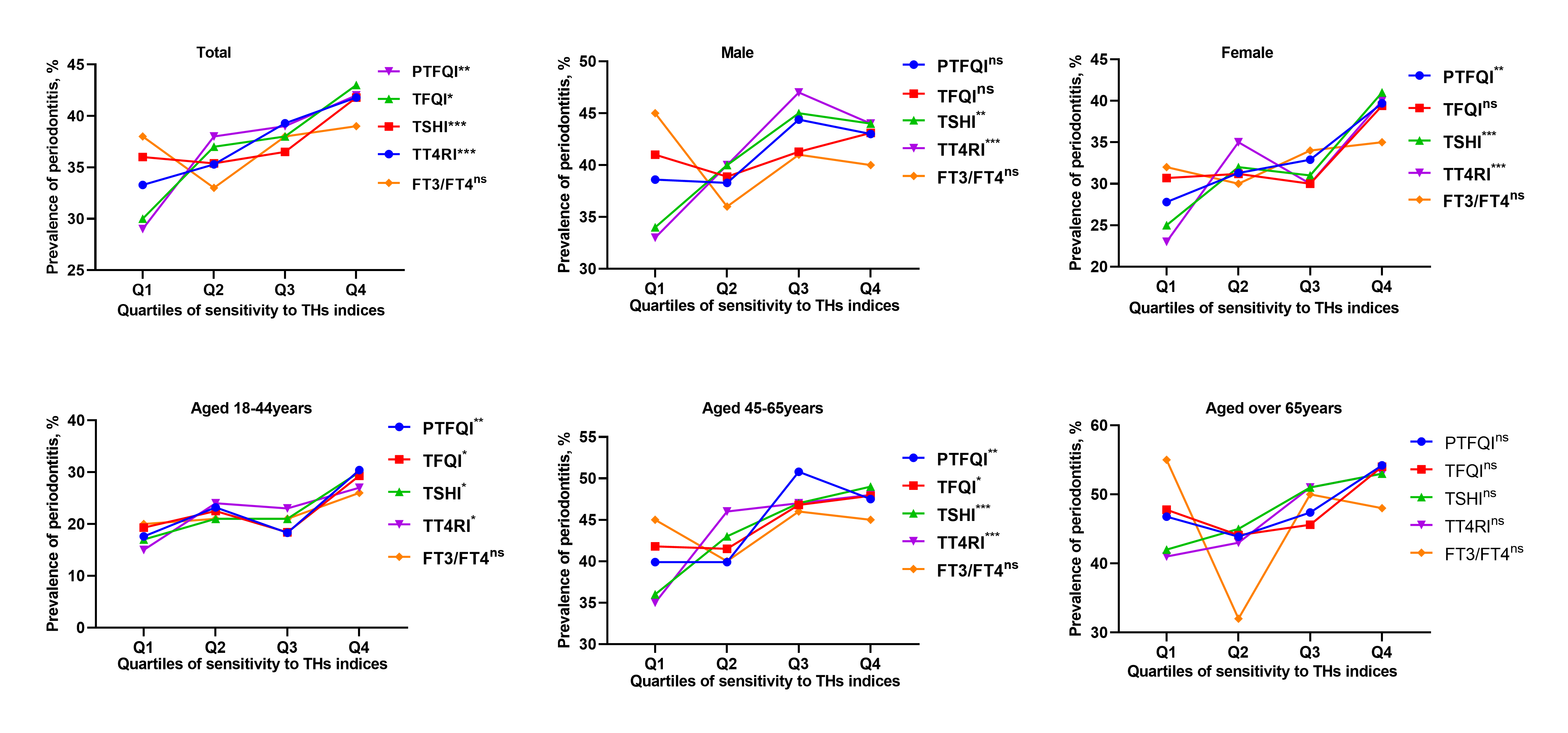

Supplement: Supplementary Figure 1 — Comparison of periodontitis prevalence among quartiles of different sensitivity to THs indices among all participants and subgroups. * P for trend <0.05, ** P for trend <0.01, *** P for trend <0.001, ns no significance. [file Image1.tif]
