# Supplementary material for: The mediating role of serum 25-hydroxyvitamin D on the association between reduced sensitivity to thyroid hormones and periodontitis in Chinese euthyroid adults
Source: Front Endocrinol (Lausanne). 2024 Oct 30;15:1456217. doi: 10.3389/fendo.2024.1456217 (PMC11557418; doi:10.3389/fendo.2024.1456217)
Supplement: Supplementary file 2 [file Table1.docx]

**Table S1** Association of sensitivity to THs indices with clinical characteristics of participants

| Characteristic | PTFQI | | TFQI | | TSHI | | TT4RI | | FT3/FT4 ratio | |
| --- | --- | --- | --- | --- | --- | --- | --- | --- | --- | --- |
|  | *r* | *p-value* | *r* | *p-value* | *r* | *p-value* | *r* | *p-value* | *r* | *p-value* |
| Age | -0.082 | ＜0.001 | -0.081 | ＜0.001 | -0.055 | 0.005 | -0.019 | 0.329 | -0.023 | 0.241 |
| WC | 0.023 | 0.249 | 0.024 | 0.221 | -0.008 | 0.687 | -0.036 | 0.071 | 0.223 | ＜0.001 |
| BMI | -0.014 | 0.481 | -0.014 | 0.476 | -0.017 | 0.384 | -0.018 | 0.372 | 0.213 | ＜0.001 |
| SBP | 0.032 | 0.113 | 0.039 | 0.047 | 0.013 | 0.509 | 0.007 | 0.733 | 0.147 | ＜0.001 |
| DBP | 0.062 | 0.002 | 0.068 | 0.001 | 0.028 | 0.154 | 0.01 | 0.626 | 0.124 | ＜0.001 |
| TG | 0.004 | 0.846 | 0.005 | 0.791 | 0.004 | 0.825 | 0.02 | 0.317 | 0.163 | ＜0.001 |
| TC | 0.018 | 0.362 | 0.014 | 0.473 | 0.049 | 0.014 | 0.068 | 0.001 | -0.039 | 0.049 |
| LDLC | 0.025 | 0.214 | 0.024 | 0.236 | 0.047 | 0.018 | 0.059 | 0.003 | -0.025 | 0.208 |
| HDLC | -0.01 | 0.619 | -0.019 | 0.33 | 0.019 | 0.346 | 0.021 | 0.296 | -0.212 | ＜0.001 |
| FBG | 0.036 | 0.067 | 0.039 | 0.046 | 0.011 | 0.600 | -0.004 | 0.854 | 0.019 | 0.342 |
| NLR | -0.024 | 0.221 | -0.019 | 0.329 | -0.052 | 0.009 | -0.066 | 0.001 | -0.069 | 0.001 |

**Table S2** Sensitivity Analysis for the association of sensitivity to THs and periodontitis risk.

|  | **Model 1** | | | **Model 2** | | | **Model 3** | | |
| --- | --- | --- | --- | --- | --- | --- | --- | --- | --- |
|  | OR | 95% CI | p-value | OR | 95% CI | p-value | OR | 95% CI | p-value |
| TFQI (+1 SD) | 1.18 | 1.06, 1.33 | 0.005 | 1.17 | 1.05, 1.31 | 0.005 | 1.17 | 1.04, 1.30 | 0.006 |
| Q1 | Ref. | | | Ref. | | | Ref. | | |
| Q2 | 1.01 | 0.76, 1.34 | 0.973 | 1.01 | 0.75, 1.34 | 0.967 | 1.02 | 0.77, 1.36 | 0.869 |
| Q3 | 1.21 | 0.91, 1.61 | 0.186 | 1.19 | 0.88, 1.58 | 0.194 | 1.22 | 0.92, 1.62 | 0.173 |
| Q4 | 1.33 | 1.01, 1.79 | 0.043 | 1.34 | 1.01, 1.79 | 0.044 | 1.34 | 1.01, 1.78 | 0.046 |
| PTFQI (+1 SD) | 1.19 | 1.07, 1.33 | 0.001 | 1.20 | 1.07, 1.33 | 0.001 | 1.19 | 1.08, 1.35 | 0.001 |
| Q1 | Ref. | | | Ref. | | | Ref. | | |
| Q2 | 0.95 | 0.71, 1.27 | 0.729 | 0.95 | 0.72, 1.27 | 0.749 | 0.96 | 0.72, 1.29 | 0.804 |
| Q3 | 1.39 | 1.05, 1.82 | 0.022 | 1.39 | 1.06, 1.86 | 0.031 | 1.40 | 1.05, 1.86 | 0.020 |
| Q4 | 1.43 | 1.07, 1.90 | 0.015 | 1.43 | 1.08, 1.91 | 0.014 | 1.42 | 1.07, 1.90 | 0.016 |
| TSHI (+1 SD) | 1.30 | 1.14, 1.48 | <0.001 | 1.30 | 1.14, 1.49 | <0.001 | 1.30 | 1.14, 1.49 | <0.001 |
| Q1 | Ref. | | | Ref. | | | Ref. | | |
| Q2 | 1.15 | 0.86, 1.54 | 0.338 | 1.15 | 0.86, 1.54 | 0.330 | 1.16 | 0.87, 1.55 | 0.311 |
| Q3 | 1.58 | 1.19, 2.10 | 0.002 | 1.58 | 1.18, 2.10 | 0.002 | 1.60 | 1.20, 2.13 | 0.001 |
| Q4 | 1.52 | 1.14, 2.03 | 0.004 | 1.53 | 1.15, 2.04 | 0.004 | 1.52 | 1.14, 2.03 | 0.004 |
| TT4RI (+1 SD) | 1.34 | 1.14, 1.57 | <0.001 | 1.34 | 1.15, 1.57 | <0.001 | 1.34 | 1.15, 1.58 | <0.001 |
| Q1 | Ref. | | | Ref. | | | Ref. | | |
| Q2 | 1.49 | 1.12, 2.00 | 0.007 | 1.50 | 1.12, 2.00 | 0.006 | 1.50 | 1.12, 2.01 | 0.006 |
| Q3 | 1.56 | 1.17, 2.09 | 0.002 | 1.56 | 1.16, 2.08 | 0.003 | 1.57 | 1.17, 2.10 | 0.002 |
| Q4 | 1.72 | 1.29, 2.30 | <0.001 | 1.72 | 1.29, 2.30 | <0.001 | 1.71 | 1.28, 2.33 | <0.001 |

OR = Odds Ratio, CI = Confidence Interval

Model 1: adjusted for sex, age, smoking, and drinking.

Model 2: adjusted for sex, age, smoking, drinking, WC, BMI, 25(OH)D, NLR.

Model 3: adjusted for sex, age, smoking, drinking, WC, BMI, 25(OH)D, NLR, diabetes, hypertension, and dyslipidemia.
